# Colo-renal Fistula in a Patient With Refractory Anemia and Recurrent Urinary Tract Infections: A Case Report and Review of the Literature

**DOI:** 10.7759/cureus.44741

**Published:** 2023-09-05

**Authors:** Emily Hillman, Hangcheng Fu, Uzoma Anele

**Affiliations:** 1 Urology, University of Louisville School of Medicine, Louisville, USA

**Keywords:** bacteremia, colostomy, urosepsis, ureteropelvic obstruction, nephrocolic fistula, colonic resection, nephrectomy, pyelonephritis, urinary fistula, renocolic fistula

## Abstract

Although rare, colo-renal fistulas pose diagnostic challenges due to their varied presentations and etiologies. Here, we present a unique case of a woman with recurrent pyelonephritis, severe anemia, and unintended weight loss, who was eventually diagnosed with a colo-renal fistula. Delayed imaging following intraoperative fluoroscopy revealed the abnormal connection between the colon and upper urinary tract. The patient underwent nephrectomy and colon resection. This case report emphasizes the need for suspicion in diagnosing such fistulas and highlights their varied management. This case adds to the literature by illustrating an unusual presentation and underscores the complexity of diagnosis and treatment.

## Introduction

Colo-renal fistulas, uncommon anomalies linking the colon and kidney, pose diagnostic difficulties due to their diverse etiologies and presentations. Inciting factors for these processes may be due to iatrogenic causes, such as upper urinary tract tumor ablation or percutaneous nephrostomy tube placement, but can also include other causes such as malignancy, trauma, infection, or chronic inflammation [1-5]. Symptoms and clinical findings associated with these fistulas are often atypical and variable. Cross-contamination of colonic and urinary tract contents may give rise to electrolyte abnormalities, diarrhea, recurrent urinary tract infections (UTIs), pneumaturia, and/or fecaluria [6].

The diagnosis of colo-renal fistula is successfully achieved using contrast-based imaging or endoscopy. Finally, management of the condition is guided by the patient’s clinical status, comorbidities, and nutritional state, with options ranging from observation to extirpation. The following case report is of a woman who had a unique presentation of idiopathic severe anemia and unintentional weight loss. She had multiple hospitalizations for sepsis consequent to emphysematous pyelitis and was discovered to have a colo-renal fistula. This case report aims to enhance the understanding of colo-renal fistulas by presenting a unique case with an atypical clinical profile. By conducting a literature review, we examine the diagnostic challenges, treatment options, and outcomes to shed light on the complexity of managing these fistulas.

## Case presentation

A 53-year-old female with a history of chronic kidney disease presented to the emergency department (ED) with several weeks of worsening fatigue associated with gross hematuria, and an unintentional weight loss of 40 pounds. Her past surgical history was notable for bilateral pyeloplasty for ureteropelvic junction (UPJ) obstruction 40 years ago. The patient had a prolonged hospital stay associated with a urinary tract infection (UTI) and severe normocytic anemia with a low hemoglobin of 5.0 g/dL, which required multiple transfusions. An extensive workup for her anemia was conducted, including an unremarkable esophagogastroduodenoscopy and colonoscopy without obvious sources of bleeding, a hemolysis panel, and a bone marrow biopsy without production abnormalities. She had no prior history of gastrointestinal abnormalities or previous colonoscopies. The patient then underwent a cystoscopy with a bilateral retrograde pyelogram that revealed severe left hydronephrosis to the UPJ, suggesting a recurrence of her prior obstruction of an atrophic left kidney. The patient was discharged following hemoglobin stabilization, and her anemia was subsequently treated with intermittent transfusions.

**Figure 1:**
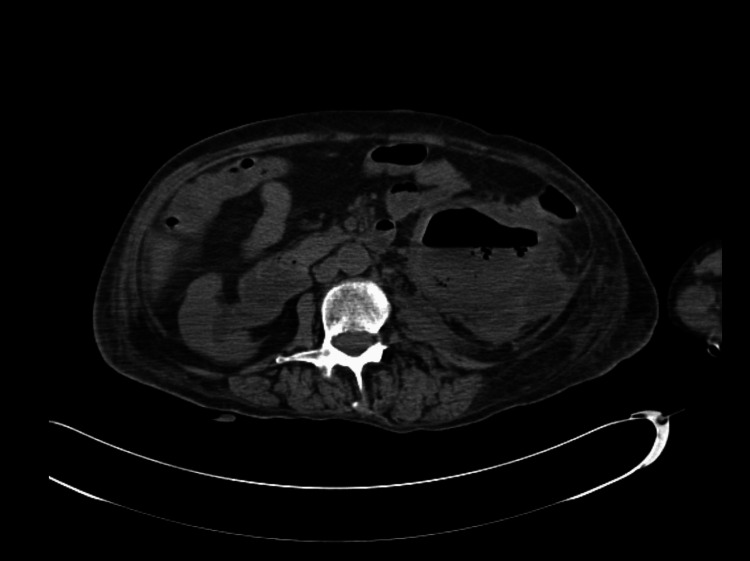
CT abdomen/pelvis without contrast at admission demonstrating air in the dilated collecting system consistent with emphysematous pyelitis

Six months later, the patient returned to the ED with altered mental status, fever, chills, dysuria, and denied diarrhea. On physical exam, she was tachycardic and hypotensive with left costovertebral angle tenderness. Pertinent laboratory values showed an elevated white blood cell count at 20,300/mm^3^, creatinine of 3.0 mg/dL, and serum lactate of 4.5 mmol/L. Urinalysis was consistent with a UTI. A CT of the abdomen/pelvis without contrast revealed emphysematous pyelitis (Figure 1).

The patient was then admitted to the intensive care unit. A subsequently performed cystoscopy with a left retrograde pyelogram showed severe left hydronephrosis with an air-fluid level without an apparent fistula or extravasation outside of the collecting system but necessitated ureteral stent placement. Despite this, the patient had a progressive clinical decline with worsening hypotension and altered mental status. She became profoundly septic with Bacteroides fragilis and Streptococcus anginosus bacteremia. The patient underwent a left percutaneous nephrostomy placement and IV antibiotics were broadened to ceftriaxone and metronidazole, which facilitated her subsequent clinical stabilization.

**Figure 2:**
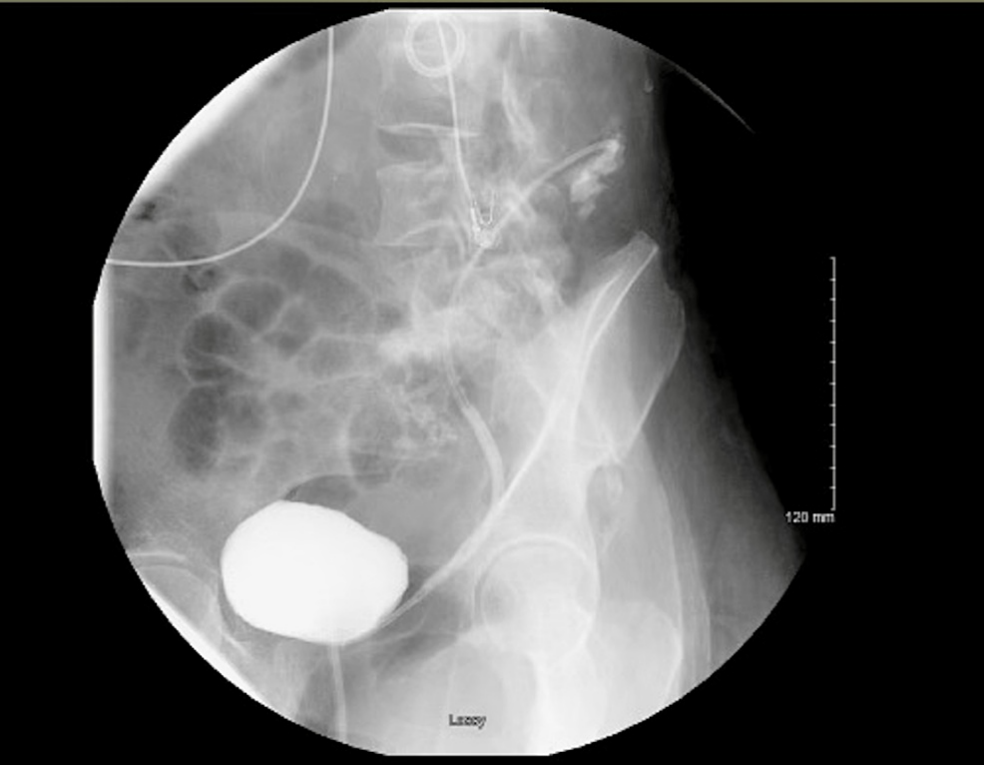
Cystogram demonstrating reflux of contrast from the bladder into the stented left upper urinary tract with opacification of the colon

Although the patient had clinically improved, the atypical presentation and persistence of Bacteroides bacteremia despite maximum drainage and antibiotic treatment raised the suspicion of a potential fistula between gastrointestinal and genitourinary tracts. A cystogram was performed and demonstrated reflux of contrast from the bladder into the stented left upper urinary tract with opacification of the colon (Figure 2).

**Figure 3:**
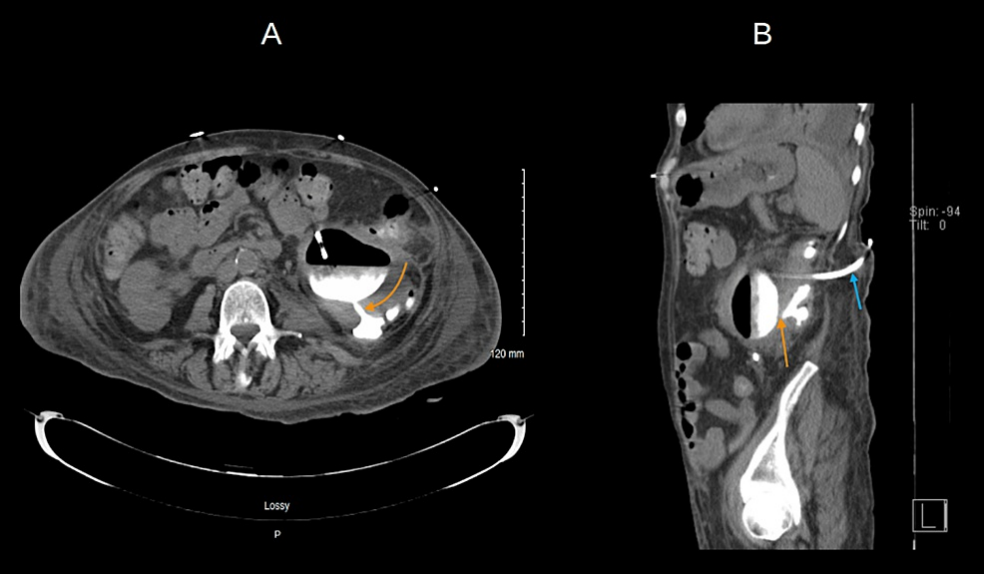
Axial and sagittal views of CT abdomen/pelvis delineating the colo-renal fistula tract A: Axial view of CT abdomen/pelvis demonstrating the renal pelvis with air and contrast. The arrow delineates the colo-renal fistula tract and below is the contrast opacifying the colon. B: Sagittal view of CT abdomen/pelvis demonstrating the nephrostomy tube tract (blue arrow), renal pelvis with air, and contrast opacifying the colon (indicated by the orange arrow).

A subsequent non-contrast CT scan demonstrated a fistulous connection with residual contrast in the left lower renal pole and proximal ureter communicating with the sigmoid colon as shown in (Figure 3).

Upon consultation with the colorectal surgery service, the patient underwent a colonoscopy, which demonstrated a profoundly ulcerated colo-renal fistula at the descending colon and hemorrhagic inflammatory tissue without malignancy on biopsy. The patient was then discharged and scheduled for a coordinated surgical intervention.

Two months later, she underwent an exploratory laparotomy with extensive adhesiolysis, left nephrectomy, left hemicolectomy, and transverse colostomy. She recovered well from the operation, and at eight months postoperative follow-up, her anemia resolved along with a 50-pound weight gain following nutritional status improvement. She eventually underwent an uncomplicated colostomy reversal that included an open colostomy takedown with end-to-side colorectal anastomosis 10 months after surgery.

## Discussion

In line with prior research, our case underscores the diagnostic challenges posed by colo-renal fistulas due to their varied presentations. Notably, our patient’s unique presentation of severe anemia and UTI without other common symptoms of pneumaturia, fecaluria, or diarrhea highlights the need for a high index of suspicion [6]. Patients may present with a variety of symptoms, including recurrent UTI, abdominal pain, hematochezia, and sepsis as indicated in Table 1.

**Table 1:** Review of pertinent case reports with respective management RCC = Renal Cell Carcinoma; CT = computed tomography; UTI = urinary tract infection; N/A = not applicable GU: Genitourinary GI: Gastrointestinal

Author/Years	Etiology	Etiology Characterization	Management	Presentations	Outcome
Mozo et al. [2]	Cryoablation for RCC	GU	Surgical repair with omental flap	Recurrent UTI, pneumaturia	Recovered
Schmit et al. [7]	Cryoablation for RCC	GU	CT-guided and endoscopic fistula repair	Recurrent UTI, pneumaturia	Recovered
Sabogal et al. [8]	Acute Lymphoblastic Leukemia	Hematologic	Colon resection, surgical repair	Recurrent UTI, abdominal pain	Recovered
Wysocki et al. [9]	Cryoablation for RCC	GU	Nephrectomy, colectomy with colostomy	Hematochezia	Recovered
Mejri et al. [10]	Tuberculosis	GU	Urgent nephrectomy	Septic Shock	Recovered
Ashfaq et al. [11]	Cryoablation for RCC	GU	Renal preservation repair, colectomy, omental flap	Urosepsis, pneumaturia, fecaluria	Recovered
Lulla et al. [12]	Staghorn Calculus	GU	N/A	Recurrent UTI	N/A
Marwah et al. [13]	Tuberculosis	GU	Nephrectomy, colectomy	Lumbar pain	Recovered
Young et al. [14]	Sigmoid Diverticulitis	GI	Colon resection, primary anastomosis	Pneumaturia, fecaluria	Recovered
Shimizu et al. [15]	Cryoablation for RCC	GU	Conservative management	Pneumaturia	Recovered
Vanderbrink et al. [16]	Cryoablation for RCC	GU	Ureteral stent placement	UTI	Recovered
Morgan et al. [17]	Cryoablation for RCC	GU	Conservative management	Pneumaturia, flank pain	Recovered

Our patient’s normocytic anemia is hypothesized to result from chronic disease caused by the chronic inflammatory state of her fistula as well as her chronic kidney disease. This was likely further compounded by nutritional deficiency due to poor gastrointestinal absorption. The patient’s previous history of congenital bilateral UPJ obstruction, bilateral pyeloplasty, and chronic left hydronephrosis also presented diagnostic challenges, particularly obfuscating the underlying nature of her recurrent UTI. Despite these non-specific presentations, suspicion of a fistula heightened after the discovery of uncharacteristic bacteremia caused by Bacteroides, a substantial component of the gastrointestinal flora [18]. Similarly, microbiology aided Mozo et al. when recurrent UTIs with urine culture-positive Escherichia (E.) coli did not improve following appropriate antibiotic therapy leading to speculation that colonic bacteria may be seeding the urinary tract [2].

Diagnostic tools like contrast imaging play a crucial role in confirming the presence of such fistulas, as demonstrated in our case. A colo-renal fistula may be readily diagnosed using retrograde or antegrade pyelography. Additionally, cross-sectional contrast imaging, particularly evaluating the urinary tract, such as CT/MR urography, may significantly aid not only in the diagnosis but also in the characterization of this disease process [19]. However, the use of intravenous contrast for these procedures is performed cautiously since renal function may be compromised at baseline as a result of the fistula. Interestingly, her initial colonoscopy during the anemia work-up was noted to be completely normal.

Characterization of the most common etiologies of colo-renal fistulas is challenging due to the rare nature of the condition. It is important to note that a fistulous tract can develop in any area affected by chronic inflammation, necrosis, or ischemia, which may occur in the setting of trauma, malignancy, or infection/abscess. A review of the literature commonly reveals underlying causes, such as interventional procedures, obstructing calculi, and inflammatory conditions like diverticulitis and Crohn’s, as well as diseases such as tuberculosis [11-14]. Interestingly, cryoablation procedures for renal cell carcinoma (RCC) appear to be the most cited cause of colo-renal fistula within the last 11 years. Shimizu et al. suspected the low temperature of cryoablation and the direct influence of the ice ball caused the occlusion of small vessels, resulting in ischemic fistulous formation [15]. Although we are unable to know with certainty, we suspect the triggering etiology for fistula formation in this present case was likely related to urinary obstruction and a chronic infectious state.

Management of colo-renal fistulas is dictated by the clinical context and functional status of the upper urinary tract. Fistula size, presence of baseline obstruction, ipsilateral kidney function, and inciting etiology may inform the method and success of the management approach. Conservative treatment may include observation with bowel rest, urinary decompression/drainage (e.g., ureteral stenting), and/or antibiotic therapy [20]. In 2007, Vanderbrink et al. showed that treatment of a colo-renal fistula after percutaneous cryoablation for RCC could be successfully resolved with an internal ureteric stent [16]. In that case, the patient had relatively normal kidney function and a fistula caused by cryoablation, which is distinctly different compared to our case. Similarly, Morgan et al. described symptom resolution of an RCC cryoablation-induced fistula following a complete non-interventional outpatient approach of prescribing antibiotics [17]. In the aforementioned cases, symptoms such as pneumaturia and flank pain occurred within three months of ablation procedures. Shimizu reported symptom resolution with patient fasting [15]. All three examples of conservative management allowed for full patient recovery.

Surgical management of colo-renal fistulas should be considered when patients present with severe sepsis, bleeding, colonic obstruction, perforation, or poor renal functionality. Due to the life-threatening complications that our patient endured from the presence of this fistula (i.e., sepsis and emphysematous pyelitis) along with the appreciably limited left renal function, the decision was made to treat her fistula surgically with extirpation. Our case, which required extensive surgical intervention, reflects the complexity of decision-making. A study by Wysocki et al. reported similar management in the context of RCC comorbidity and diabetes as a precautionary measure instead of attempting a repair [9]. However, renal preservation can also be feasible in certain situations. In the case reported by Ashfaq et al., the patient had failed conservative measures, but renal preservation was possible following laparoscopic fistula takedown, diverting colostomy, and renal repair with an omental flap [11]. Although the above invasive approaches have been more widely described, successful fistulous tract closure has been demonstrated using percutaneous CT-guided and endoscopic intervention [7]. The long-term outcomes of such procedures warrant further investigation and a consensus on optimal management strategy.

## Conclusions

In summary, this case provides insight into the intricate diagnosis and management of colo-renal fistulas. Our patient's unique presentation highlighted the diagnostic challenges and demonstrated the significance of contrast imaging. The case underscores the need for heightened awareness, particularly when faced with atypical presentations. Successful management requires a balanced consideration of conservative and surgical approaches. Further research and larger cohort studies are essential to establish standardized guidelines for the optimal diagnosis and treatment of colo-renal fistulas.
